# Aspergiloid I, an unprecedented spirolactone norditerpenoid from the plant-derived endophytic fungus *Aspergillus* sp. YXf3

**DOI:** 10.3762/bjoc.10.282

**Published:** 2014-11-17

**Authors:** Zhi Kai Guo, Rong Wang, Wei Huang, Xiao Nian Li, Rong Jiang, Ren Xiang Tan, Hui Ming Ge

**Affiliations:** 1 Laboratory of Biology and Genetic Resources of Tropical Crops, Ministry of Agriculture, Institute of Tropical Bioscience and Biotechnology, Chinese Academy of Tropical Agricultural Sciences, Haikou 571101, People’s Republic of China; 2 Hainan Academy of Ocean and Fisheries Sciences, Haikou, Hainan 570203, People’s Republic of China; 3 Institute of Functional Biomolecules, State Key Laboratory of Pharmaceutical Biotechnology, Nanjing University, Nanjing 210093, People’s Republic of China; 4 State Key Laboratory of Phytochemistry and Plant Resources in West China, Kunming Institute of Botany, Chinese Academy of Sciences, Kunming 650204, People’s Republic of China

**Keywords:** *Aspergillus*, endophytic fungus, *Ginkgo biloba*, natural product, norditerpenoid, Trichocomaceae

## Abstract

An unusual C_18_ norditerpenoid, aspergiloid I (**1**), was isolated from the culture broth of *Aspergillus* sp. YXf3, an endophytic fungus derived from *Ginkgo biloba*. Its structure was unambiguously established by analysis of HRMS–ESI and spectroscopic data, and the absolute configuration was determined by low-temperature (100 K) single crystal X-ray diffraction with Cu Kα radiation. This compound is structurally characterized by a new carbon skeleton with an unprecedented 6/5/6 tricyclic ring system bearing an α,β-unsaturated spirolactone moiety in ring B, and represents a new subclass of norditerpenoid, the skeleton of which is named aspergilane. The hypothetical biosynthetic pathway for **1** was also proposed. The cytotoxic, antimicrobial, anti-oxidant and enzyme inhibitory activities of **1** were evaluated.

## Introduction

**Figure 1:**
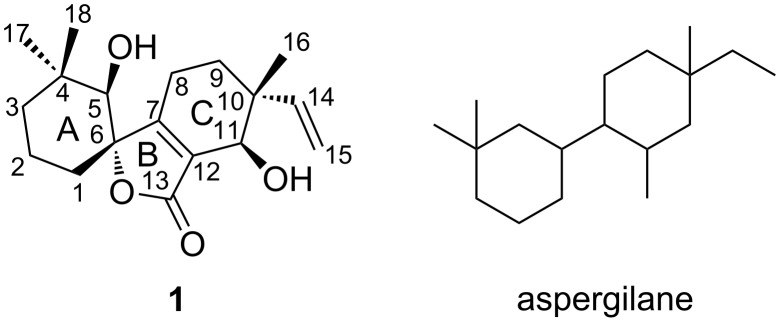
Structure of aspergiloid I (**1**) and its novel skeleton.

Plant-derived fungi, which have drawn considerable attention from natural product chemists, have been proved to be a rich source of bioactive natural compounds [1–2]. Recently, a wide variety of biologically active and structurally unique metabolites were isolated from these types of microorganisms [3–6], demonstrating their promise as a source of novel and/or bioactive natural products. Our previous chemical investigation of the bioactive secondary metabolites produced by the endophytic *Aspergillus* sp. YXf3 associated with *Ginkgo biloba* led to the isolation of new *p*-terphenyls and novel types of diterpenoids including pimarane-type diterpenoids (sphaeropsidins A and B, aspergiloids D and E), a cleistanthane-type diterpenoid (aspergiloid C), and norcleistanthane-type diterpenoids (asergiloids A, B, and F–H), many of which were reported from this microorganism for the first time [7–9]. Interestingly, sphaeropsidins A and B were also discovered from both *Aspergillus chevalieri* and phytopathogenic fungus *Sphaeropsis sapinea*, displaying anti-gram-positive bacterial, antiviral, antiprotozoal and phytotoxic activity [10–12]. We further focused on the fractionation containing the minor terpenoid constituents with characteristic signals for terminal vinyl group detected by ^1^H NMR from the liquid fermentation broth of *Aspergillus* sp. YXf3 and isolated a novel norditerpenoid, namely, aspergiloid I (**1**) (Figure 1). Herein, we report the production, isolation, structure characterization, and biological activity of **1**, a rare spirolactone metabolite with a novel carbon skeleton.

## Results and Discussion

A large-scale culture broth of *Aspergillus* sp. YXf3 was extracted with EtOAc and separated by a combination of column chromatographic methods. A preliminary survey of the fractionation by ^1^H NMR and LC–MS revealed the presence of the molecule we eventually named aspergiloid I (**1**), which had a low-resolution mass ([M + Na]^+^ at *m*/*z* 329) spectrum that did not match any previously isolated compounds, prompted us to purify it further. The obtained fermented broth (~45 L) was extracted four times with EtOAc (v/v, 1:1) to afford a brown crude extract (9.1 g). Subsequent fractionation by silica gel column chromatography (CC), octadecylsilyl (ODS) CC, Sephadex LH-20 and semi-preparative reversed-phase HPLC yielded **1** (4.3 mg).

Aspergiloid I (**1**) was isolated as a colorless lamellar crystal, with the molecular formula C_18_H_26_O_4_ (6 double-bond equivalents) as derived from the ESI high-resolution mass spectrometry ([M + Na]^+^ at *m*/*z* 329.1729, calculated 329.1723) and NMR data (Table 1). The IR spectrum exhibited absorptions at 3649 (hydroxy group) and 1735 cm^−1^ (carbonyl group). The ^1^H NMR spectrum (acquired in DMSO-*d*_6_) displayed signals of a terminal vinyl group at δ_H_ 5.72 (H-14), 4.99 (H-15α) and 4.96 (H-15β), two oxygenated methine protons (δ_H_ 3.76, 2.81), ten aliphatic protons [δ_H_ 2.56, 2.25, 1.96, 1.63, 1.61, 1.51 (2H), 1.42, 1.20, 1.11], three aliphatic methyl groups (δ_H_ 1.03, 1.02, 0.86) and two hydroxy groups (δ_H_ 5.21, 5.04). The ^13^C NMR and DEPT spectra revealed that **1** contained 18 carbons, attributable to three methyl groups (δ_C_ 23.4, 24.5, 29.2), five aliphatic methylenes (δ_C_ 18.1, 23.7, 28.4, 28.5, 31.8), one olefinic methylene (δ_C_ 113.2), two oxygenated methines (δ_C_ 76.9, 64.7), one olefinic methine (δ_C_ 142.9), and six non-protonated carbon atoms (one of which was identified as lactone group) (δ_C_ 35.1, 40.0, 88.6, 126.6, 170.9, 171.6). These data show that **1** has two double bonds and one carbonyl which require three degrees of unsaturation, thus, **1** must also contain three rings.

**Table 1:** ^1^H and ^13^C NMR spectroscopic data for aspergiloid I (**1**).

Position	δ_C_^a^	δ_H_^a^ (mult, *J* in Hz)	δ_C_^b^	δ_H_^b^ (mult, *J* in Hz)

1	28.5, CH_2_	1.96, td (13.5, 4.5, H_α_); 1.20, br d (14.0, H_β_)	28.9	2.00, td (13.5, 4.0, H_α_); 1.43, br d (13.5, H_β_)
2	18.1, CH_2_	1.63, m (H_α_); 1.51, m (overlap, H_β_)	18.2	1.87, qt (13.5, 4.0, H_α_); 1.61, dq (13.5, 4.0, H_β_)
3	31.8, CH_2_	1.51, m (overlap, H_α_); 1.11, br d (13.5, H_β_)	32.0	1.53, td (13.5, 4.0, H_α_); 1.32, br d (13.5, H_β_)
4	35.1, C		35.3	
5	76.9, CH	2.81, d (7.0)	79.0	3.09, br s
5-OH		5.21, d (7.0)		
6	88.6, C		88.8	
7	170.9, C		171.5	
8	23.7, CH_2_	2.56, ddd (20.0, 13.0, 4.5, H_α_); 2.25, dd (20.0, 4.5, H_β_)	23.7	2.78, dtd (20.0, 6.7, 1.5, H_α_); 2.34, dtd (20.0, 6.0, 1.5, H_β_)
9	28.4, CH_2_	1.61, td (13.0, 6.0, H_α_); 1.42, dd (13.0, 6.0, H_β_)	30.5	1.71, tdd (13.5, 7.5, 6.0, H_α_); 1.64, dt (13.5, 6.0, H_β_)
10	40.0, C		39.9	
11	64.7, CH	3.76, d (6.5)	67.4	4.31, s
11-OH		5.04, d (6.5)		
12	126.6, C		126.9	
13	171.6, C		172.1	
14	142.9, CH	5.72, dd (17.5, 11.0)	143.7	5.80, dd (17.2, 11.0)
15	113.2, CH_2_	4.99, dd (17.5, 1.5, H_α_); 4.96, dd (11.0, 1.5, H_β_)	113.1	5.06, d (17.2, H_α_); 5.05, d (11.0, H_β_)
16	23.4, CH_3_	1.02, s	20.1	1.08, s
17	24.5, CH_3_	1.03, s	24.6	1.17, s
18	29.2, CH_3_	0.86, s	28.6	0.97, s

^a^Acquired in DMSO-*d*_6_ (125 MHz and 500 MHz). ^b^Acquired in CDCl_3_ (125 MHz and 500 MHz).

**Figure 2:**
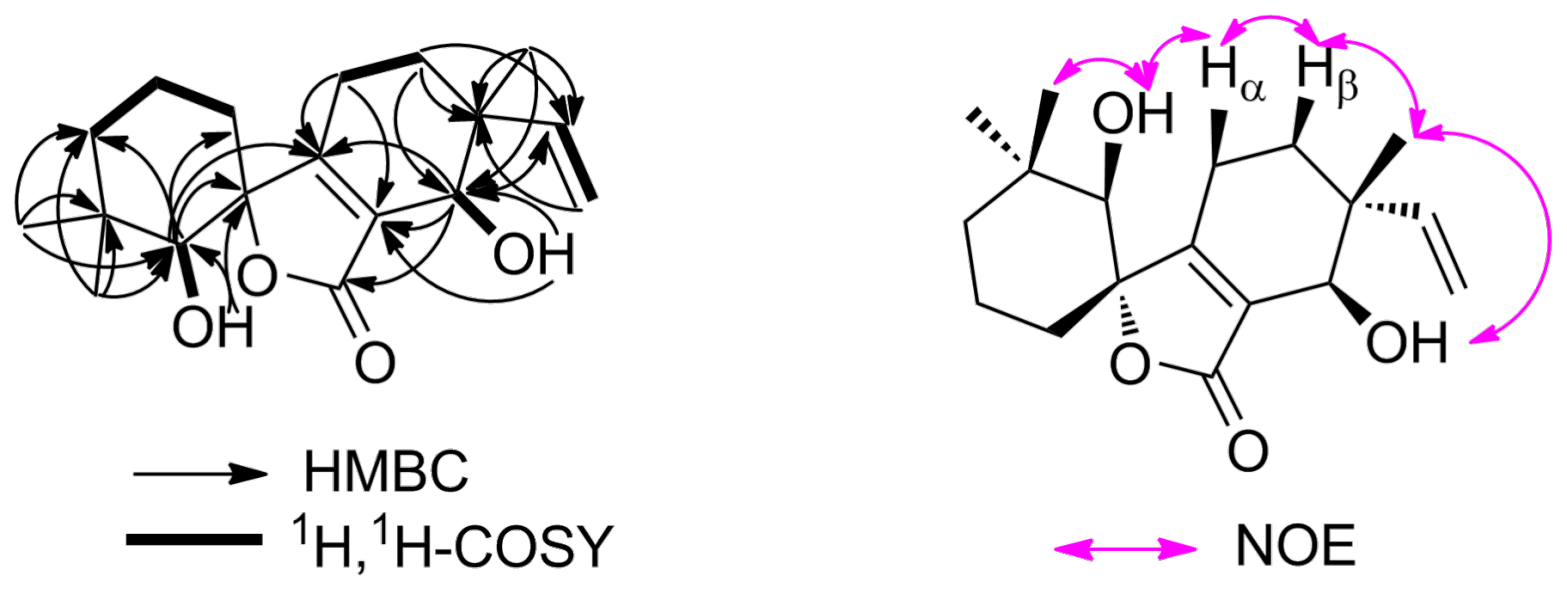
Selected ^1^H,^1^H-COSY and HMBC correlations and key NOEs observed for **1**.

The gross structure of **1** was initially deduced by comprehensive analysis of its 1D and 2D NMR data. The ^13^C NMR and HSQC spectra of **1** allowed all protons to be assigned to their respective carbons. The ^1^H,^1^H three-bond couplings from H-1 to H-3 observed in the COSY experiment established a spin system from C-1 to C-3 (Figure 2). The COSY correlation between H-8 and H-9 revealed C-8 to C-9 connectivity. A terminal vinyl moiety H-14/H_2_-15 was also confirmed by ^1^H,^1^H-COSY correlations. The hydroxy group (δ_H_ 5.21) attached to C-5 and the other hydroxy group (δ_H_ 5.04) attached to C-11 were identified by the ^1^H,^1^H couplings (acquired in DMSO-*d*_6_) with H-5 (δ_H_ 2.81), and H-11 (δ_H_ 3.76), respectively. HMBC correlations from two singlet methyl groups’ protons H_3_-17 and H_3_-18 to C-3, C-4, and C-5 indicate that C-17 and C-18 were located on the same quaternary carbon C-4, which was connected by C-3 and C-5. HMBC correlations from the hydroxy proton (δ_H_ 5.21) to C-5, and C-6 (acquired in DMSO-*d*_6_), and from H-5 to C-1, C-6, and C-7 (acquired in CDCl_3_) assigned the connectivity of the C-6 to C-1, C-5, and C-7. The other singlet methyl group, C-16, and the terminal vinyl group (C-14–C-15), were also located on the same quaternary carbon, C-10, which was flanked by C-9 and C-11 deduced from the HMBC correlations from H_2_-9 to C-10, C-11, and C-14, from H-11 to C-14, from H_2_-15 to C-10, and from the methyl protons (H_3_-16; δ_H_ 1.02) to C-10, C-11, and C-14. The HMBC correlations from the hydroxy proton (δ_H_ 5.04) to C-12, from H-11 to C-7, C-12, and C-13 indicated that C-12 was linked to C-7, C-11, and C-13 to form an α,β-unsaturated enone fragment. HMBC correlations from H_2_-8 to C-7 and C-12 secured the connectivity of the C-8 to C-7. The connectivity of C-6 to the ketone carbon C-13 through an ester linkage, which was also supported by the downfield chemical shift of C-6 (δ_C_ 88.6) completed the structure of 6/5/6 tricyclic spirolactone. The relative configuration of aspergiloid I (**1**) could be determined by NOESY correlations. The NOESY spectrum showed correlations of OH-5 with H_3_-18 and H-8α, of H-9β with H-8α and H_3_-16, and of H_3_-16 with OH-11, indicating that OH-5, H_3_-16, and OH-11 were on the same plane, while the relative configuration of the chiral center C-6 was further confirmed by a NOESY correlation of H-1α with H-8β. Therefore, the structure of compound **1** was elucidated as shown in Figure 1, representing a new type of carbon skeleton in the norditerpenoid family.

**Figure 3:**
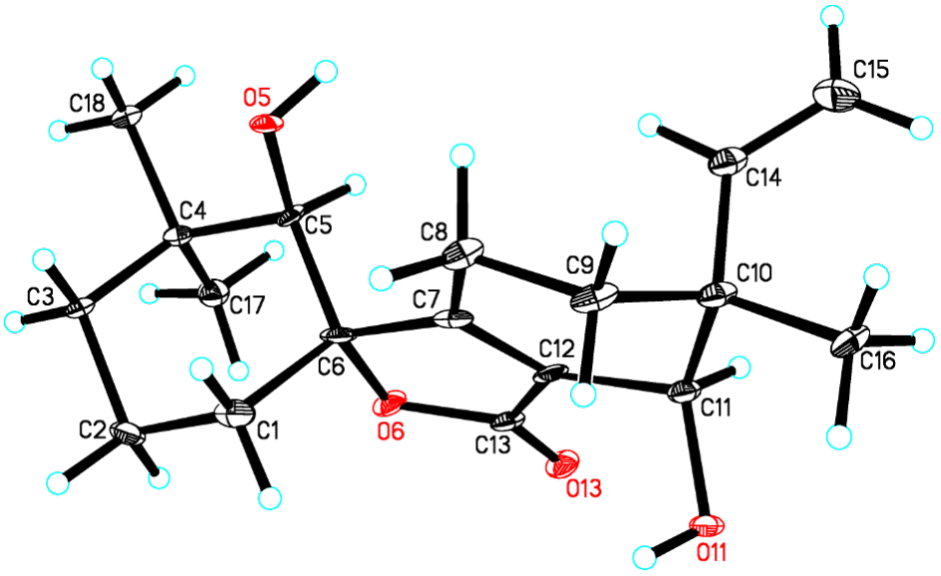
X-ray single-crystal structure of **1**.

The structure of **1** was further confirmed by a low-temperature (100 K) single-crystal X-ray diffraction experiment, which is shown in Figure 3. As compound **1** has a relatively high percentage of oxygen, it shows enough anomalous dispersion of Cu Kα radiation and allows to determinate the absolute stereochemistry with the Hooft parameter 0.17(15) for 992 Bijvoet pairs by single-crystal X-ray diffraction experiment [13]. Therefore, the absolute configurations of the chiral centers in **1** were established as 5*R*, 6*S*, 10*R*, 11*R*. This compound is structurally characterized by a new carbon skeleton with an unprecedented 6/5/6 tricyclic ring system bearing an α,β-unsaturated spirolactone moiety in ring B. The skeleton, tentatively named aspergilane, represents a new subclass of norditerpenoids.

**Scheme 1:**
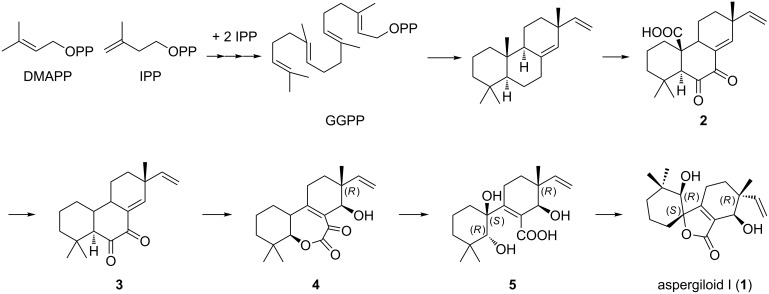
Proposed biosynthetic pathway of **1**.

The endophytic fungus *Aspergillus* sp. YXf3 can produce different types of diterpenoids, including pimarane-type diterpenoids (sphaeropsidin A and B, aspergiloid D and E), cleistanthane type-diterpenoid (aspergiloid C), and norcleistanthane-type diterpenoids (aspergiloid A, B, and F–H) [7–9], and the “aspergilane”-type norditerpenoid aspergiloid I. A plausible biogenetic relation is given in Scheme S1 (Supporting Information File 1) for the formation of these diterpenoids. Here the hypothetical pimarane compound **2**, the hemiketal lactone ring-opening product of aspergiloid E, was proposed as the most probable biosynthetic intermediate. As shown in Scheme 1, we suggest the biosynthesis of **1** starts from the classical diterpene precursor geranylgeranyl diphosphate [14], and intermediate **2** undergoes decarboxylation to form **3** through Baeyer–Villiger oxidation to form the 7-membered lactone **4**, then hydrolyzation, decarboxylation and lactonization to finally give aspergiloid I (**1**).

Aspergiloid I (**1**) was evaluated for its cytotoxicity against eleven human cancer cell lines, K562 myeloid leukemia, SH-SY5Y neuroblastoma, SGC-7901 gastric adenocarcinoma, HepG2, SMMC-7721 hepatocellular carcinoma, A549 lung cancer, MCF-7, MDA-MB-231 breast cancer, HCT116, SW480 colon cancer, HT29 colorectal cancer. However, no significant activity was detected (IC_50_ > 50 μM). It also displayed no anti-oxidant, and acetylcholinesterase (AChE), α-glucosidase, and topoisomerase IIα inhibitory activities at a concentration of 50 μg/mL. Antimicrobial activities against a variety of plant pathogenic bacteria (*Xanthomonas oryzae* pv. *oryzae* Swings, *Xanthomonas oryzae* pv. *oryzicola* Swings, *Acidovorax avenae* subsp. *Citrulli*, *Erwinia amylovora*, *Pseudomonas syringae* pv. *Lachrymans*, *Clavibacter michiganense* subsp. *Sepedonicus*, and *Pectobacterium carotovorum* subsp. *carotovorum*) and fungi (*Rhizoctonia solani* Kühn, *Rhizotonia cerealis* van der Hoeven, *Phytophthora capsici* Leonian, *Fusarium moniliforme* Sheld, *Alternaria solani* Jones et Grout, *Sclerotinia sclerotiorum* de Bary, *Fusarium graminearum* Schw., *Fusarium coeruleum* Sacc., and *Botrytis cinerea* Pers.) were tested. Aspergiloid I (**1**) showed no antibacterial or antifungal activity at a concentration of 20 μg/mL. It also had no antifungal activity against *Candida albicans* (ATCC 10231) or *Fusarium oxysporum* f. sp. cubense race 4 at concentrations as high as 20 mg/mL.

## Conclusion

In summary, guided by ^1^H NMR detection, we isolated and characterized a novel norditerpenoid, aspergiloid I (**1**), from the liquid fermentation broth of the endophytic *Aspergillus* sp. YXf3 associated with *Ginkgo biloba*. This compound is structurally characterized by a new carbon skeleton with an unprecedented 6/5/6 tricyclic ring system bearing a α,β-unsaturated spirolactone moiety, and represents a new subclass of norditerpenoid, the skeleton of which is named aspergilane. Chemical investigation of *Aspergillus* sp. YXf3 revealed it is a pluripotent fungus which can produce different types of novel interesting metabolites, including *p*-terphenyls, pimarane, cleistanthane, norcleistanthane-type diterpenoids [7], and the “aspergilane”-type norditerpenoid **1**. It is possible to propose that **1** is biosynthetically derived from hypothetical intermediate pimarane compound **2**, the hemiketal lactone ring-opening product of aspergiloid E. In biological tests, **1** showed no cytotoxic, antimicrobial, anti-oxidant, acetylcholinesterase (AChE), α-glucosidase, and topoisomerase IIα inhibitory activities. In order to perform more biological assays for this unusual norditerpenoid, further scale-up isolation is in progress.

## Experimental

### General experimental procedures

The melting point was measured on a Beijing Taike X-5 stage apparatus and reported without correction. The optical rotation was recorded using a Rudolph Autopol III polarimeter. The UV spectrum was obtained on a Hitachi U-3000 spectrophotometer. The CD spectrum was measured on a JASCO J-810 spectrometer, and the IR spectrum (KBr) was obtained on a Nexus 870 FTIR spectrometer. NMR data were acquired using a Bruker AVANCE III-500 NMR spectrometer at 500 MHz for ^1^H NMR and 125 MHz for ^13^C NMR. The chemical shifts were given in δ (ppm) and referenced to the solvent signal (DMSO-*d*_6_, δ_H_ 2.50, δ_C_ 39.5; CDCl_3_, δ_H_ 7.26, δ_C_ 77.1) as the internal standard, and coupling constants (*J*) are reported in Hz. The high resolution mass measurement was conducted on an Agilent 6210 TOF LC–MS spectrometer. Silica gel (200–300 mesh; Qingdao Marine Chemical Factory, Qingdao, China) and Sephadex LH-20 gel (Pharmacia Biotech, Sweden) were used for column chromatography (CC). Semipreparative HPLC was conducted on a Waters ODS (250 × 4.6 mm, 5 μm) on a Hitachi HPLC system consisting of a L-7110 pump (Hitachi) and a L-7400 UV–vis detector (Hitachi). All other chemicals used in this study were of analytical grade.

### Fungal material, cultivation, extraction and isolation

The fungal strain *Aspergillus* sp. YXf3 was isolated by one of the authors (Z.K.G.) from a healthy leaf of *Ginkgo biloba* collected in the campus of Nanjing University (Nanjing, P. R. China), in October 2008 [7]. The strain was cultured on MEA (consisting of 20 g/L malt extract, 20 g/L sucrose, 1 g/L peptone, 20 g/L agar and deionized water) at 28 °C for 5 days. Agar plugs were used to inoculate in 1000 mL Erlenmeyer flasks, each containing 300 mL of ME liquid media. Fermentation was carried out on a rotary shaker (140 rpm) at 26 °C for 13 days in 1000 mL Erlenmeyer flasks. Mycelia were separated by filtration and the obtained fermented broth (about 45 L) was extracted four times with EtOAc (v/v, 1:1) to afford a brown crude extract (9.1 g), which was then fractionated by silica gel (91 g) CC (8 × 100 cm) eluted with a gradient of CHCl_3_–MeOH (v/v 100:0, 100:1, 100:2, 100:4, 100:8, 100:16, 0:100, each 1200 mL) to produce seven fractions. Fraction 2 (1.52 g; CHCl_3_–MeOH, 100:1) was separated on a ODS column (5 × 50 cm) with a gradient of MeOH–H_2_O (v/v 30:70, 40:60, 50:50, 60:40, 70:30, 80:20, 100:0, each 500 mL) to give seven subfractions. The fourth subfraction (108.6 mg; MeOH–H_2_O, 60:40) was further isolated by Sephadex LH-20 CC eluting with MeOH and purified by semipreparative reversed-phase HPLC to yield **1** (4.3 mg) (64% MeOH in water; *t*_R_ = 17.94 min).

Aspergiloid I (**1**): colorless lamellar crystals; mp 181.0**–**184.9 °C; [α]^28^_D_ −19.5 (*c* 0.2, MeOH); UV (MeOH) λ_max_ (log ε): 220.0 (3.97) nm; CD (MeOH): Δε = 214 (+5.30), 242 (−8.23), 283 (+0.04), 374 (−0.54), 399 (+0.50), 411 (−0.14), 471 (+1.69), 488 (−0.53), 500 (+1.19) nm; IR (KBr) ν_max_ 3649, 1735, 1700, 1652, 1558, 1540, 1508, 1457, 816 cm^−1^; ^1^H and ^13^C NMR data, see Table 1; HRMS–ESI (*m*/*z*): [M + Na]^+^ calcd for C_18_H_26_O_4_Na, 329.1723; found, 329.1729.

### X-ray crystallographic analysis of **1**

Colorless crystal of **1** was obtained by crystallizing from a solution of 2 mL methanol with two drops of distilled water. The single crystal X-ray diffraction data were collected at 100 K with Cu Kα radiation (λ = 1.54178 Å) on a Bruker APEX DUO CCD diffractometer, equipped with an Oxford Cryostream 700+ cooler. Structures were solved using the program SHELXS-97 [15], and refined anisotropically by full-matrix least-squares on *F*^2^ using SHELXL-97. The absolute configurations were determined by computation of the Hooft parameter [13], in all cases yielding a probability of 1.000 that the reported configuration is correct. Crystal data: C_18_H_26_O_4_, *M* = 306.39, orthorhombic, *a* = 6.3349(2) Å, *b* = 11.1090(3) Å, *c* = 23.3643(6) Å, α = 90.00°, β = 90.00°, γ = 90.00°, *V* = 1644.25(8) Å^3^, *T* = 100(2) K, space group *P*212121, *Z* = 4, μ(Cu Kα) = 0.694 mm^−1^, 7387 reflections measured, 2729 independent reflections (*R*_int_ = 0.0603). The final *R*_1_ values were 0.0901 (*I* > 2σ(*I*)). The final *wR*(*F*^2^) values were 0.2603 (*I* > 2σ(*I*)). The final *R*_1_ values were 0.1025 (all data). The final *wR*(*F*^2^) values were 0.2816 (all data). The goodness of fit on *F*^2^ was 1.126. The Hooft parameter is 0.17(15) for 992 Bijvoet pairs. Crystallographic data for the structure of aspergiloid I (**1**) have been deposited with the Cambridge Crystallographic Data Centre (deposition no. CCDC 985728). These data can be obtained free of charge via http://www.ccdc.cam.ac.uk/data_request/cif, or by contacting The Cambridge Crystallographic Data Centre, 12 Union Road, Cambridge CB21EZ, UK; fax: +44-1223-336-033; or desposit@ccdc.cam.ac.uk.

### Biological assays

Cytotoxic activity against 11 human cancer cell lines, K562 myeloid leukemia, SH-SY5Y beuroblastoma, SGC-7901 gastric adenocarcinoma, HepG2, SMMC-7721 hepatocellular carcinoma, A549 lung cancer, MCF-7, MDA-MB-231 breast cancer, HCT116, SW480 colon cancer, HT29 colorectal cancer, were evaluated with the MTT assay [16–17]. Antimicrobial activities against a variety of plant pathogenic bacteria (*Xanthomonas oryzae* pv. *oryzae* Swings, *Xanthomonas oryzae* pv. *oryzicola* Swings, *Acidovorax avenae* subsp. *Citrulli*, *Erwinia amylovora*, *Pseudomonas syringae* pv. *Lachrymans*, *Clavibacter michiganense* subsp. *Sepedonicus*, and *Pectobacterium carotovorum* subsp. *carotovorum*) and fungi (*Rhizoctonia solani* Kühn, *Rhizotonia cerealis* van der Hoeven, *Phytophthora capsici* Leonian, *Fusarium moniliforme* Sheld, *Alternaria solani* Jones et Grout, *Sclerotinia sclerotiorum* de Bary, *Fusarium graminearum* Schw., *Fusarium coeruleum* Sacc., *Botrytis cinerea* Pers., and *Fusarium oxysporum* f. sp. cubense race 4), and *Candida albicans* (ATCC 10231), and the antioxidant, acetylcholinesterase (AChE) , α-glucosidase, and topoisomerase IIα inhibitory activities were performed in accordance with the primary literature [18–20].

## Supporting Information

File 1 1D, 2D NMR spectra, HRMS–ESI, and the X-ray crystallographic structure of **1**.
