# Supplementary material for: Aspergiloid I, an unprecedented spirolactone norditerpenoid from the plant-derived endophytic fungus Aspergillus sp. YXf3
Source: Beilstein J Org Chem. 2014 Nov 17;10:2677–82. doi: 10.3762/bjoc.10.282 (PMC4273245; doi:10.3762/bjoc.10.282)
Supplement: File 1 — 1D, 2D NMR spectra, HRMS–ESI, and the X-ray crystallographic structure of 1. [file Beilstein_J_Org_Chem-10-2677-s001.pdf]

Supporting Information

for

Aspergiloid I, an unprecedented spirolactone norditerpenoid from the plant-derived endophytic fungus *Aspergillus* sp. YXf3

Zhi Kai Guo[1], Rong Wang[2], Wei Huang[3], Xiao Nian Li[4], Rong Jiang[3], Ren Xiang Tan[3*], Hui Ming Ge[3*]

Address: [1]Laboratory of Biology and Genetic Resources of Tropical Crops, Ministry of Agriculture, Institute of Tropical Bioscience and Biotechnology, Chinese Academy of Tropical Agricultural Sciences, Haikou 571101, People's Republic of China, [2]Hainan Academy of Ocean and Fisheries Sciences, Haikou, Hainan 570203, People's Republic of China, [3]Institute of Functional Biomolecules, State Key Laboratory of Pharmaceutical Biotechnology, Nanjing University, Nanjing 210093, People's Republic of China and [4]State Key Laboratory of Phytochemistry and Plant Resources in West China, Kunming Institute of Botany, Chinese Academy of Sciences, Kunming 650204, People's Republic of China

Email: Ren Xiang Tan - rxtan@nju.edu.cn; Hui Ming Ge - hmge@nju.edu.cn

*Corresponding author

1D, 2D NMR spectra, HRMS–ESI, and the X-ray crystallographic structure of **1**

Table of contents

S1. X-ray crystallographic analysis of **1**

Scheme S1. Plausible biosynthetic pathway of apergiloid E-I and sphaeropsidin A-F

Figure S1. ^{1}H NMR (500 MHz, DMSO-d_6) spectrum of compound **1**

Figure S2. ^{13}C NMR (125 MHz, DMSO-d_6) spectrum of compound **1**

Figure S3. DEPT spectra of compound **1** in DMSO-d_6

Figure S4. HSQC spectrum of compound **1** in DMSO-d_6

Figure S5. ^{1}H-^{1}H COSY spectrum of compound **1** in DMSO-d_6

Figure S6. HMBC spectrum of compound **1** in DMSO-d_6

Figure S7. Enlarged HMBC spectrum (δ_C 180-160 ppm, δ_H 6.1-0.6 ppm) of compound **1** in DMSO-d_6

Figure S8. NOESY spectrum of compound **1** in DMSO-d_6

Figure S9. ^{1}H NMR (500 MHz, $CDCl_3$) spectrum of compound **1**

Figure S10. ^{13}C NMR (125 MHz, $CDCl_3$) spectrum of compound **1**

Figure S11. HMQC spectrum of compound **1** in $CDCl_3$

Figure S12. ^{1}H-^{1}H COSY spectrum of compound **1** in $CDCl_3$

Figure S13. HMBC spectrum of compound **1** in $CDCl_3$

Figure S14. Enlarged HMBC spectrum (δ_C 177-167 ppm, δ_H 5.0-0.9 ppm) of compound **1** in $CDCl_3$

Figure S15. HRESI-MS spectrum of compound **1** in MeOH

Figure S16. ^{1}H NMR (500 MHz, acetone-d_6) of compound **6**

Figure S17. HRESIMS spectrum of compound **6**

Figure S18. ^{1}H NMR (500 MHz, acetone-d_6) of compound **7**

Figure S19. HRESIMS spectrum of compound **7**

S1. The X-ray crystallographic structure of **1**

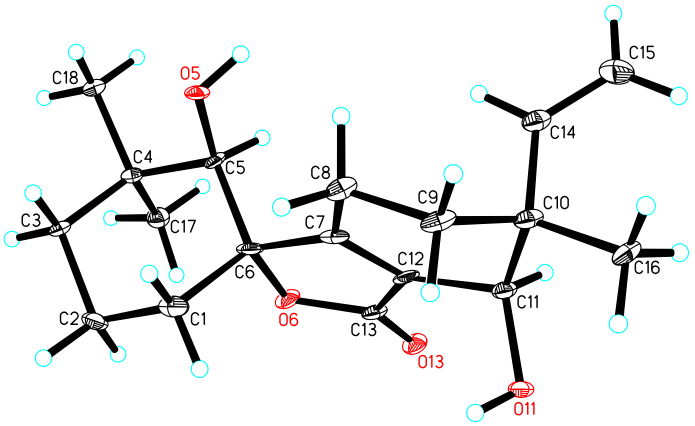

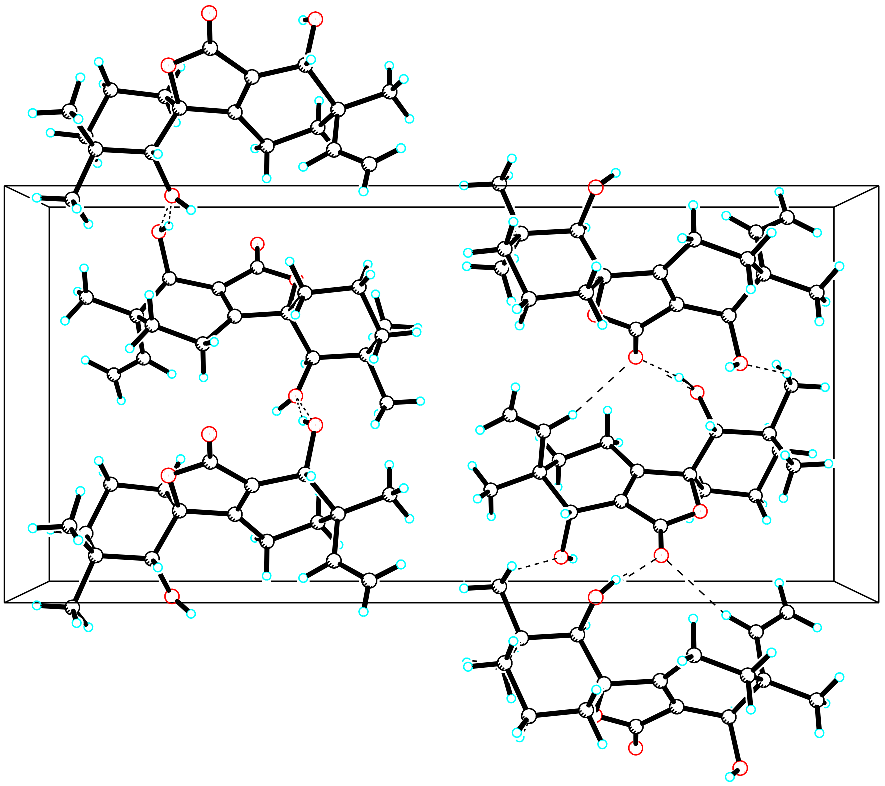

Scheme S1. Plausible biosynthetic pathway of apergiloid **A-I** and sphaeropsidin **A-F**

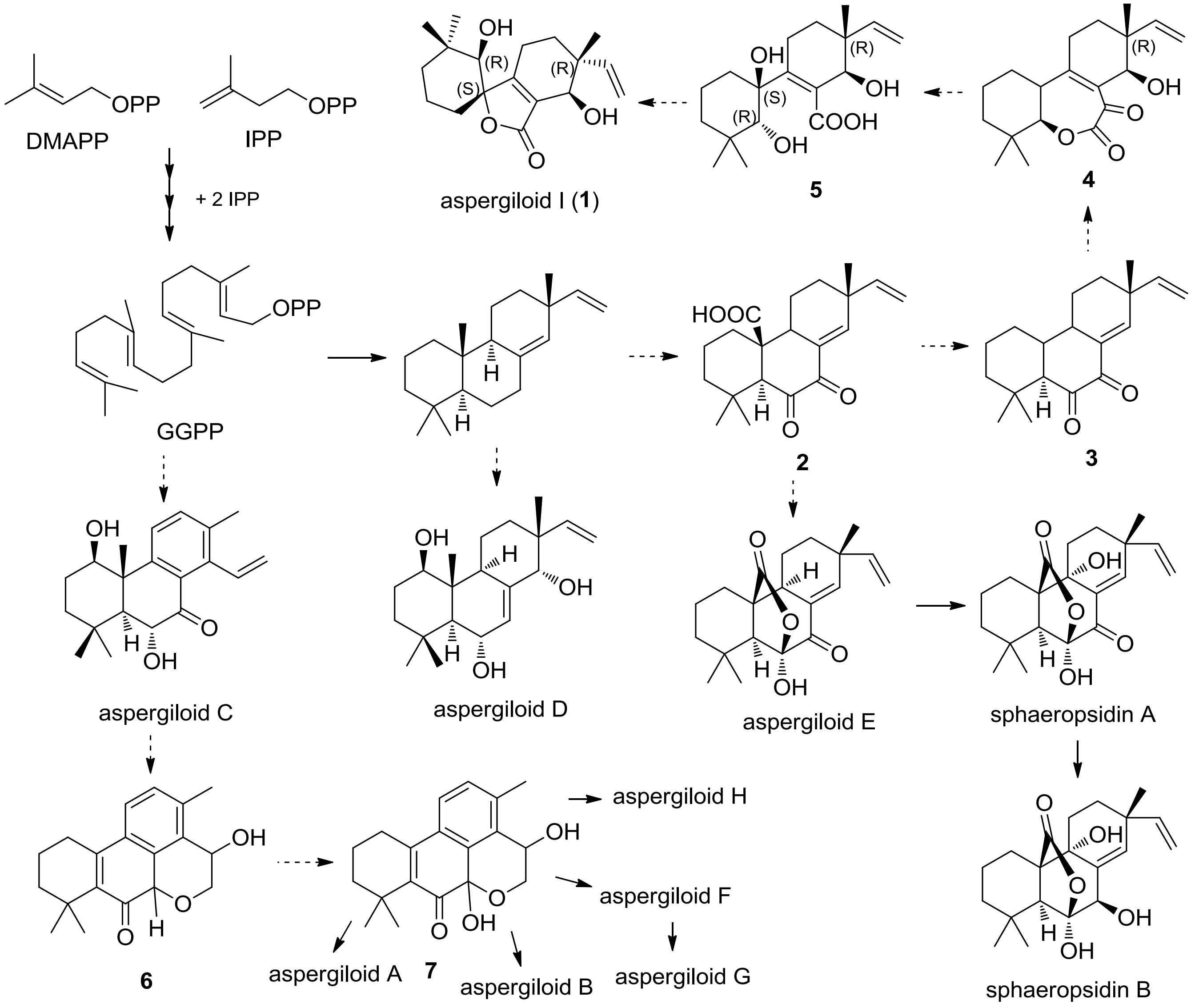

References:

1 Guo, Z. K.; Yan, T.; Guo, Y.; Song, Y. C.; Jiao, R. H.; Tan, R. X.; Ge, H. M. *J. Nat. Prod.* **2012**, *75*, 15–21.

2 Yan, T.; Guo, Z. K.; Jiang, R.; Wei, W.; Wang, T.; Guo, Y.; Song, Y. C.; Jiao, R. H.; Tan, R. X.; Ge, H. M. *Planta Med*. **2013**, *79*, 348–352.

3 Guo, Z. K.; Liu, S. B.; Ma S. Nat. Prod. Res. Dev. **2013**, *25*, 778–781.

4 Ellestad, G. A. ; Kunstmann, M. P. ; Mirando, P.; Morton, G. O. *J. Am. Chem. Soc.* **1972**, *94*, 6206–6208.

5 Evidente, A.; Sparapano, L.; Fierro, O.; Bruno, G.; Giordano, F.; Motta, A. *Phytochemistry*, **1997**, *45*, 705–713.

6 Evidente, A.; Sparapano, L.; Bruno, G.; Motta, A. *Phytochemistry*, **2002**, *59*, 817–823.

7 Dewick, P. M. *Nat. Prod. Rep.* **2002**, *19*, 181–122.

Figure S1. ^{1}H NMR (500 MHz, DMSO-d_6) spectrum of compound **1**

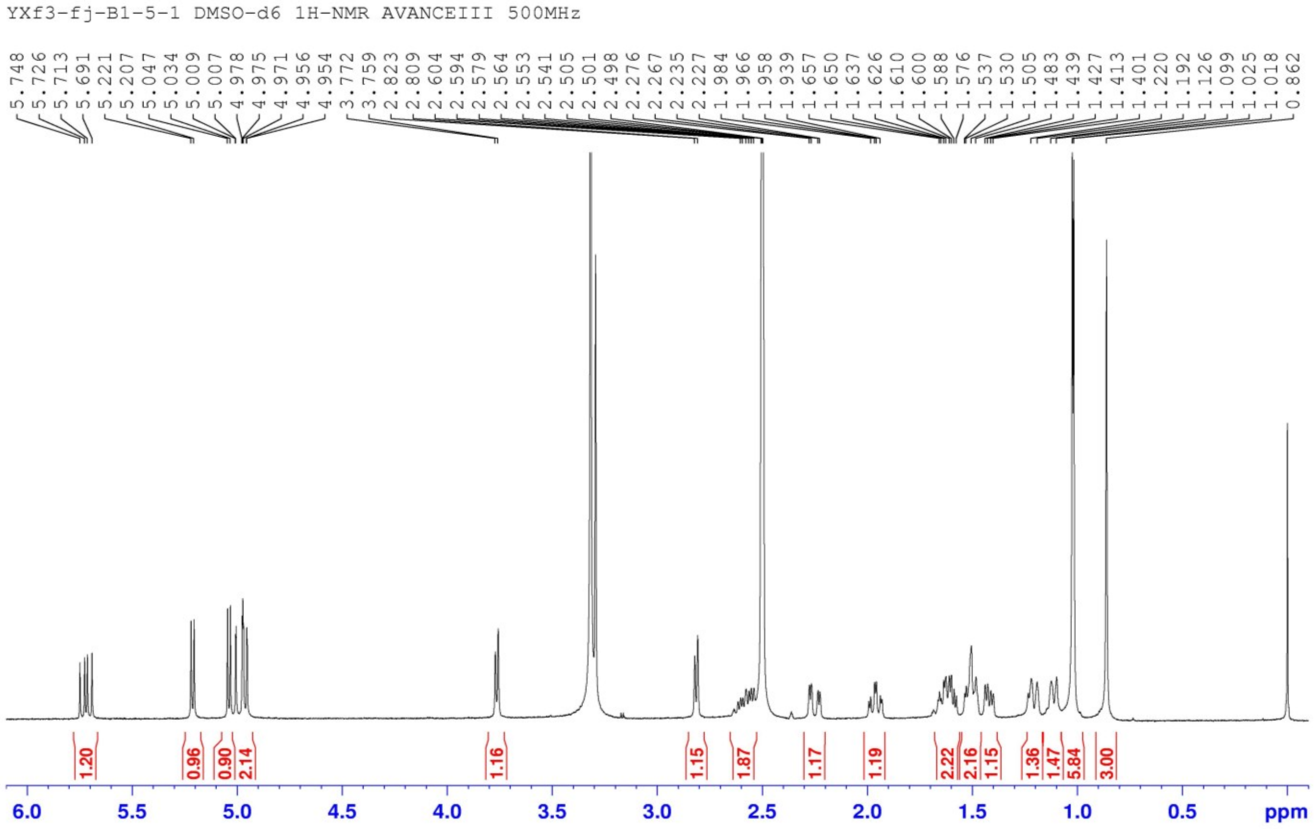

Figure S2. ^{13}C NMR (125 MHz, DMSO-d_6) spectrum of compound **1**

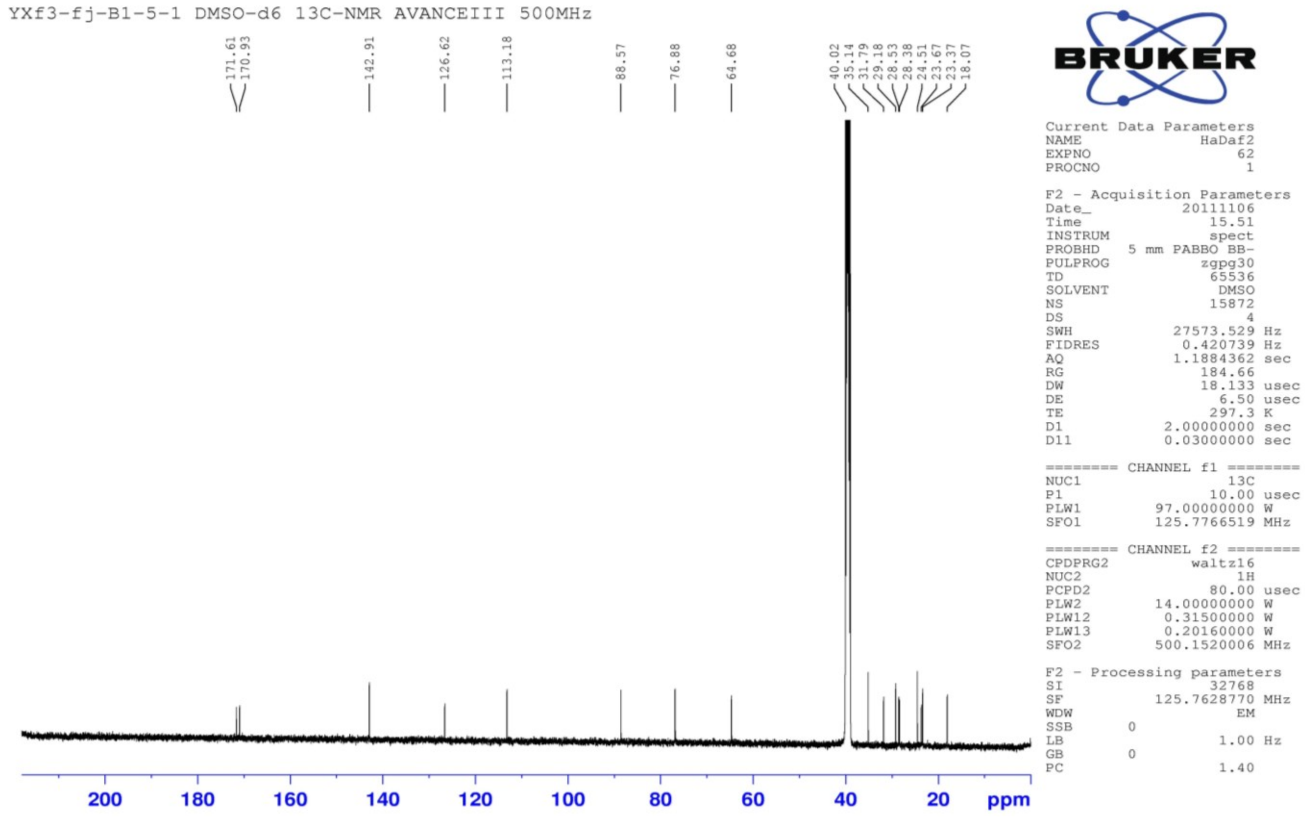

Figure S3. DEPT spectra of compound **1** in DMSO-d_6

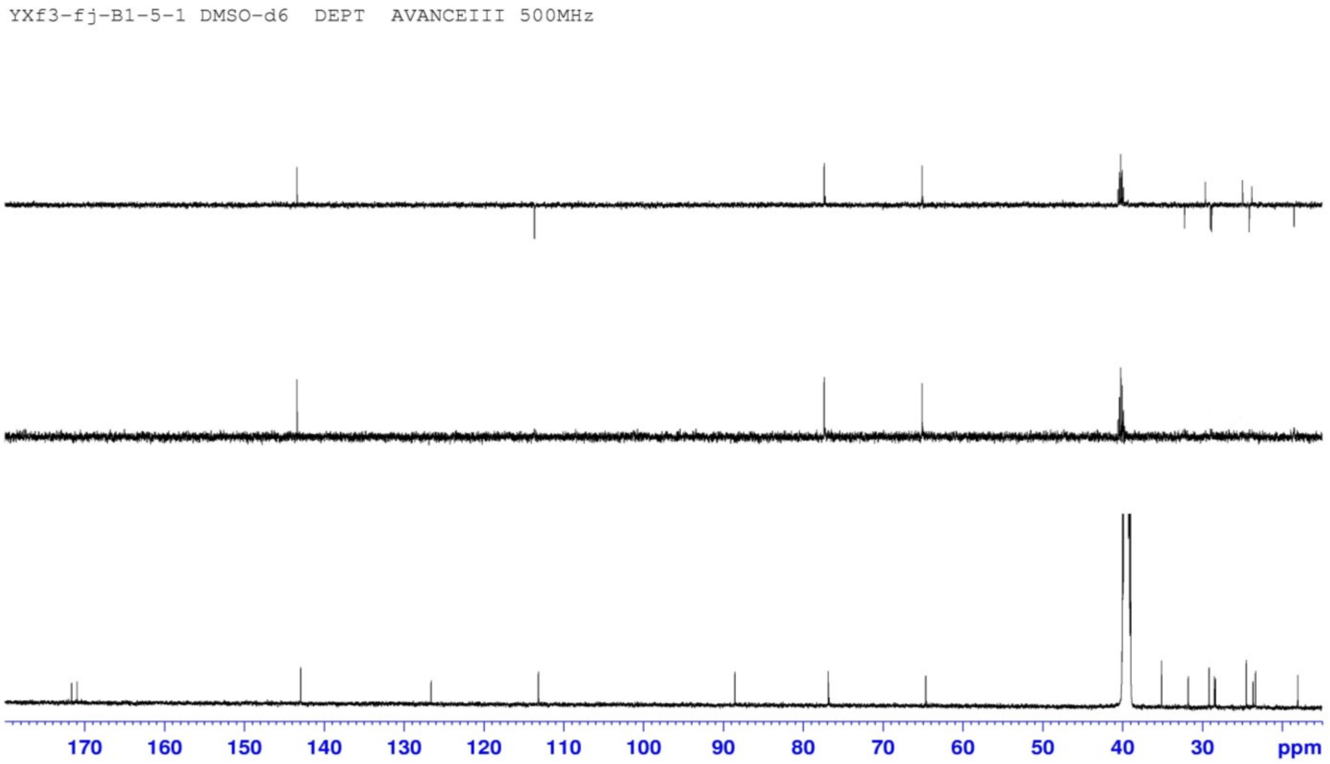

Figure S4. HSQC spectrum of compound **1** in DMSO-d_6

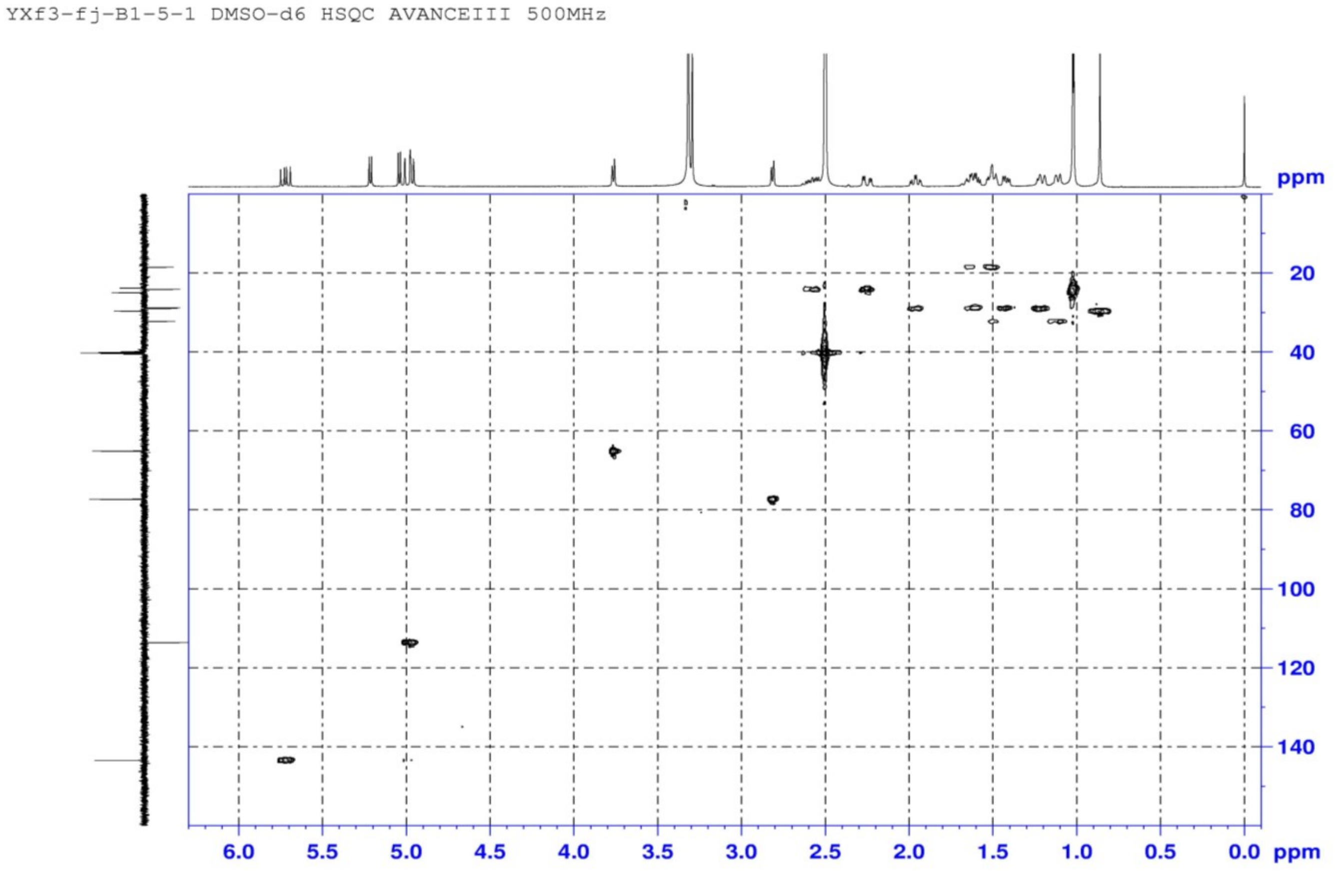

Figure S5. ^{1}H-^{1}H COSY spectrum of compound **1** in DMSO-d_6

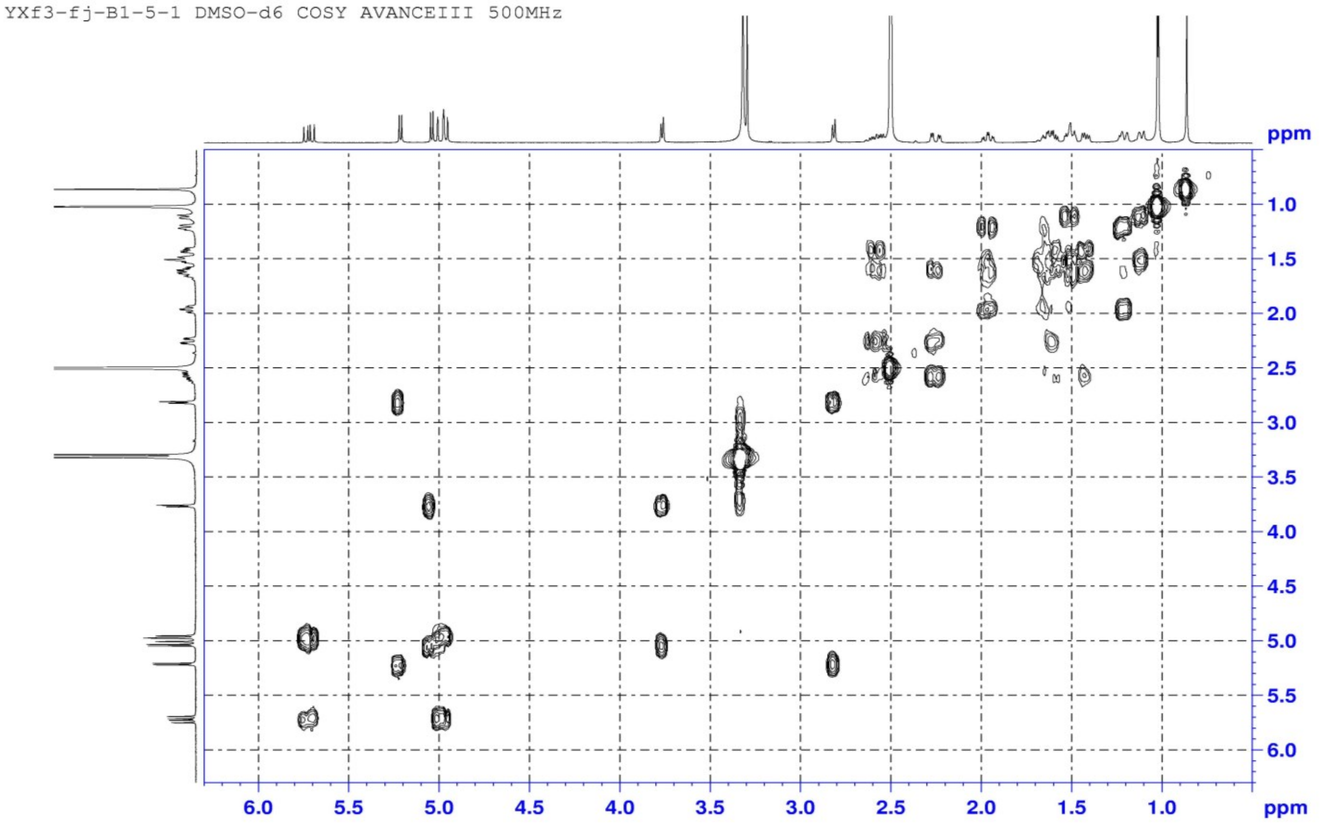

Figure S6. HMBC spectrum of compound **1** in DMSO-d_6

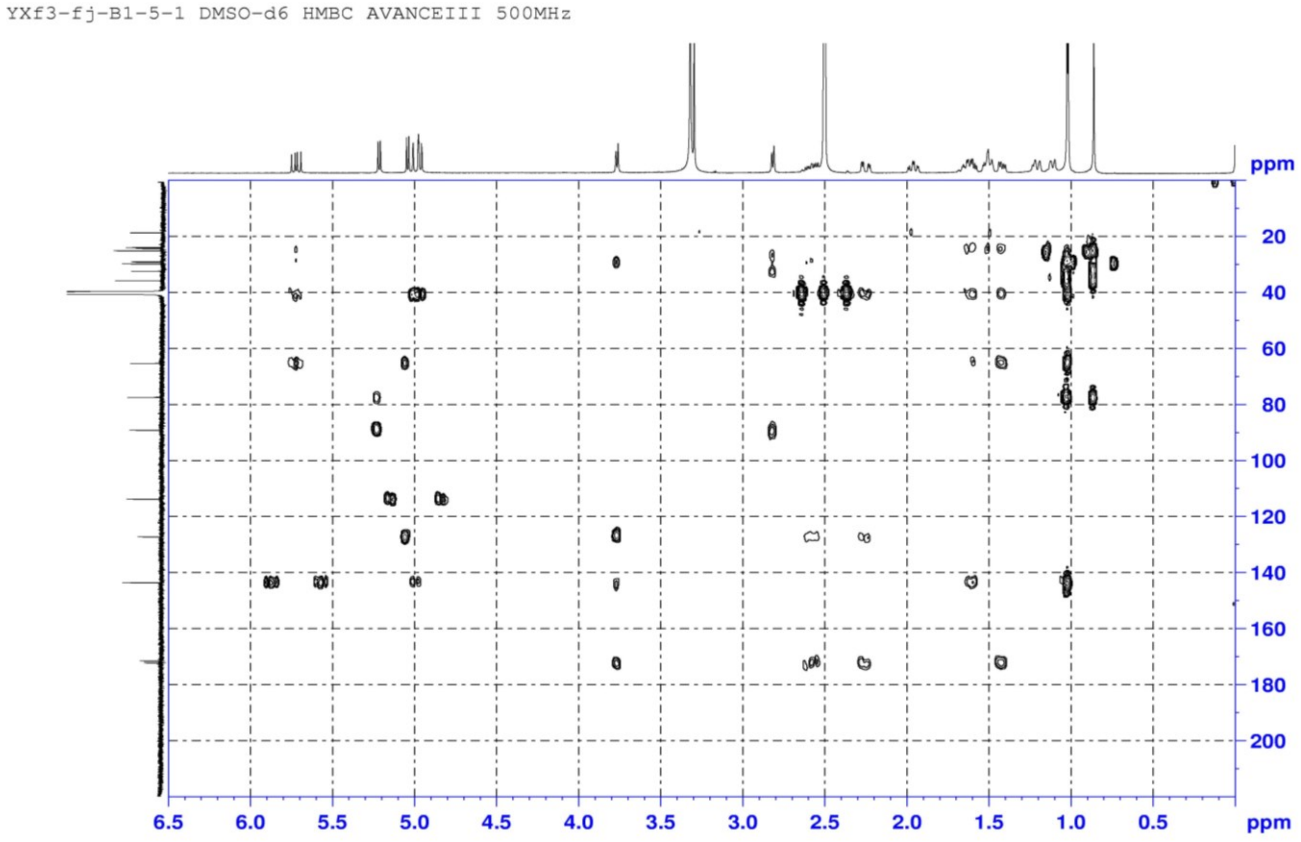

Figure S7. Enlarged HMBC spectrum (δ_C 180-160 ppm, δ_H 6.1-0.6 ppm) of compound **1** in DMSO-d_6

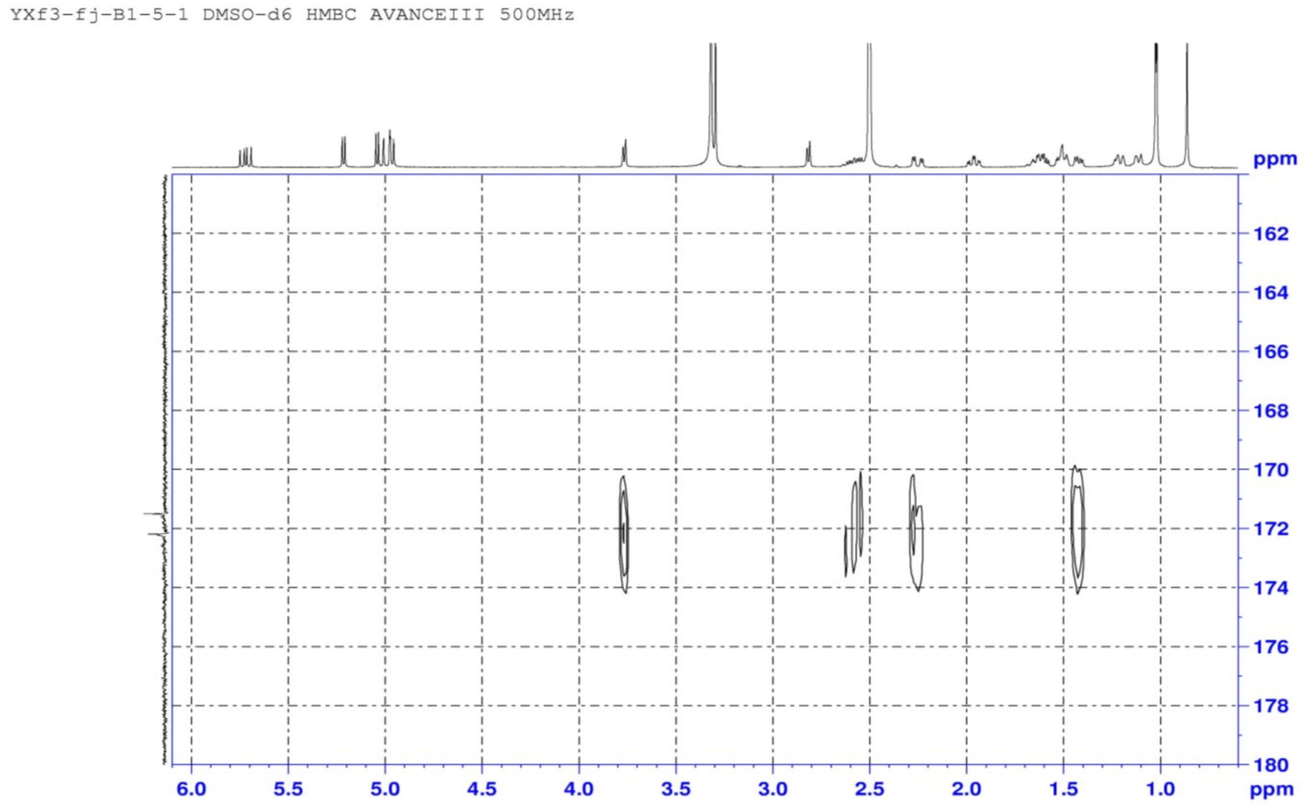

Figure S8. NOESY spectrum of compound **1** in DMSO-d_6

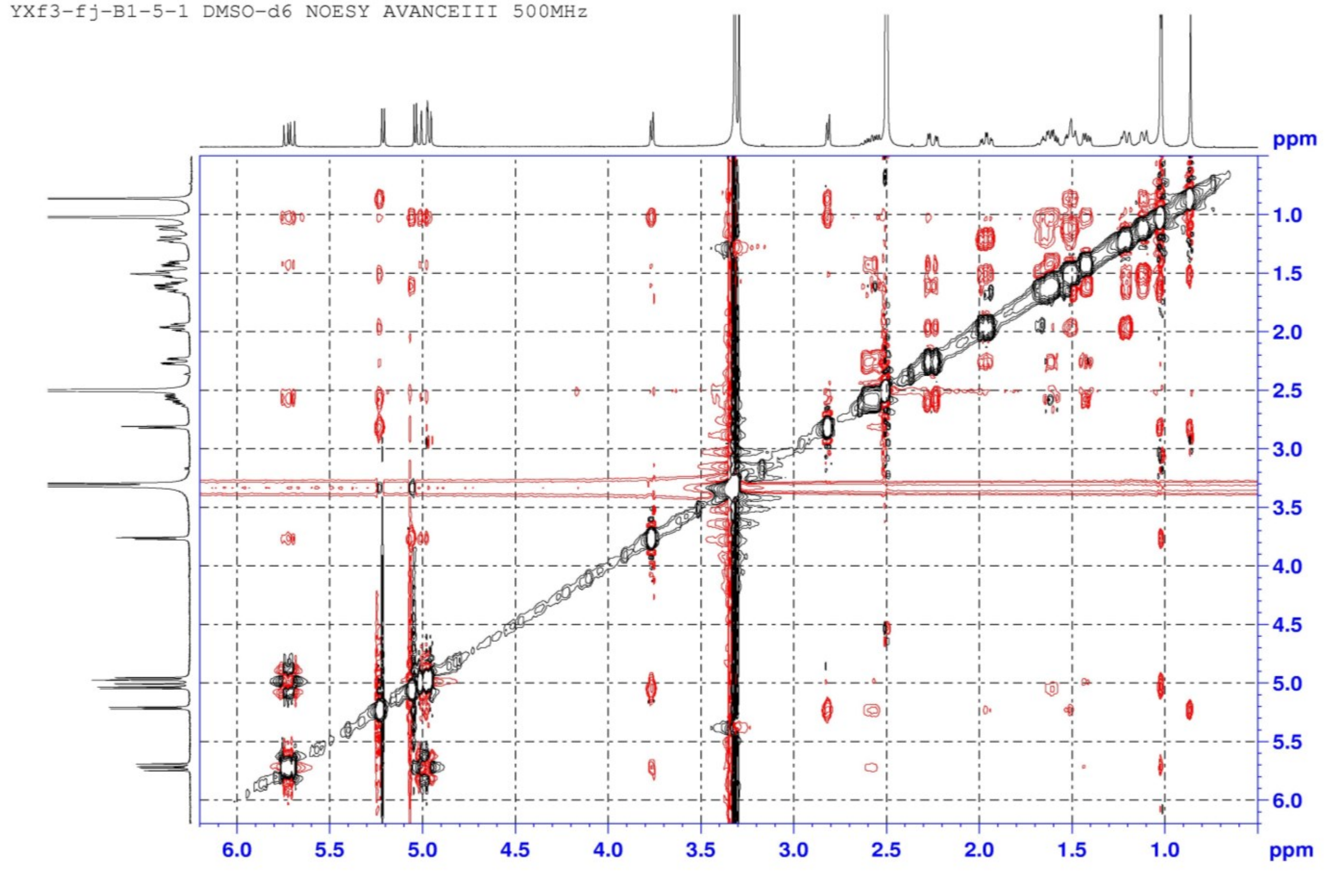

Figure S9. ^{1}H NMR (500 MHz, $CDCl_3$) spectrum of compound **1**

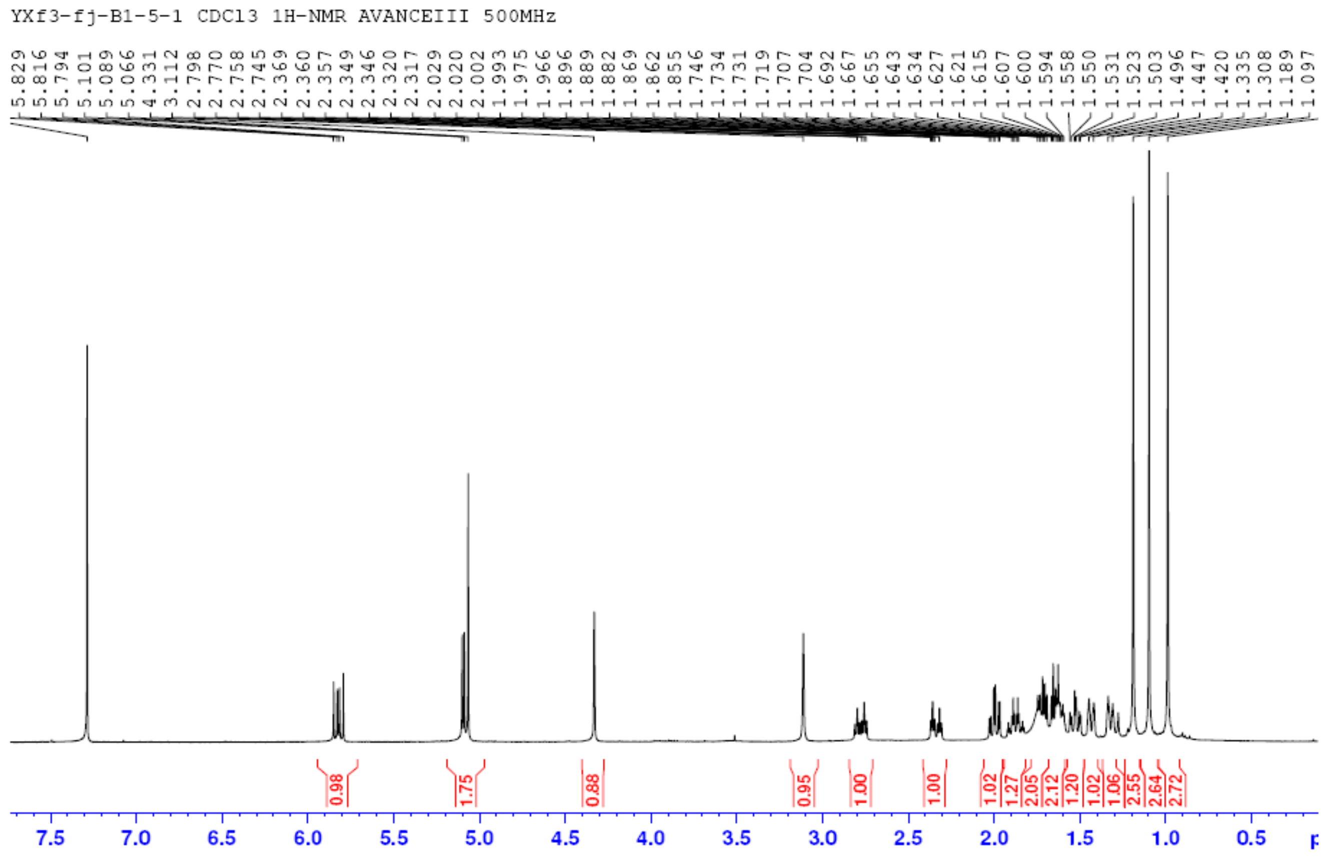

Figure S10. ^{13}C NMR (125 MHz, $CDCl_3$) spectrum of compound **1**

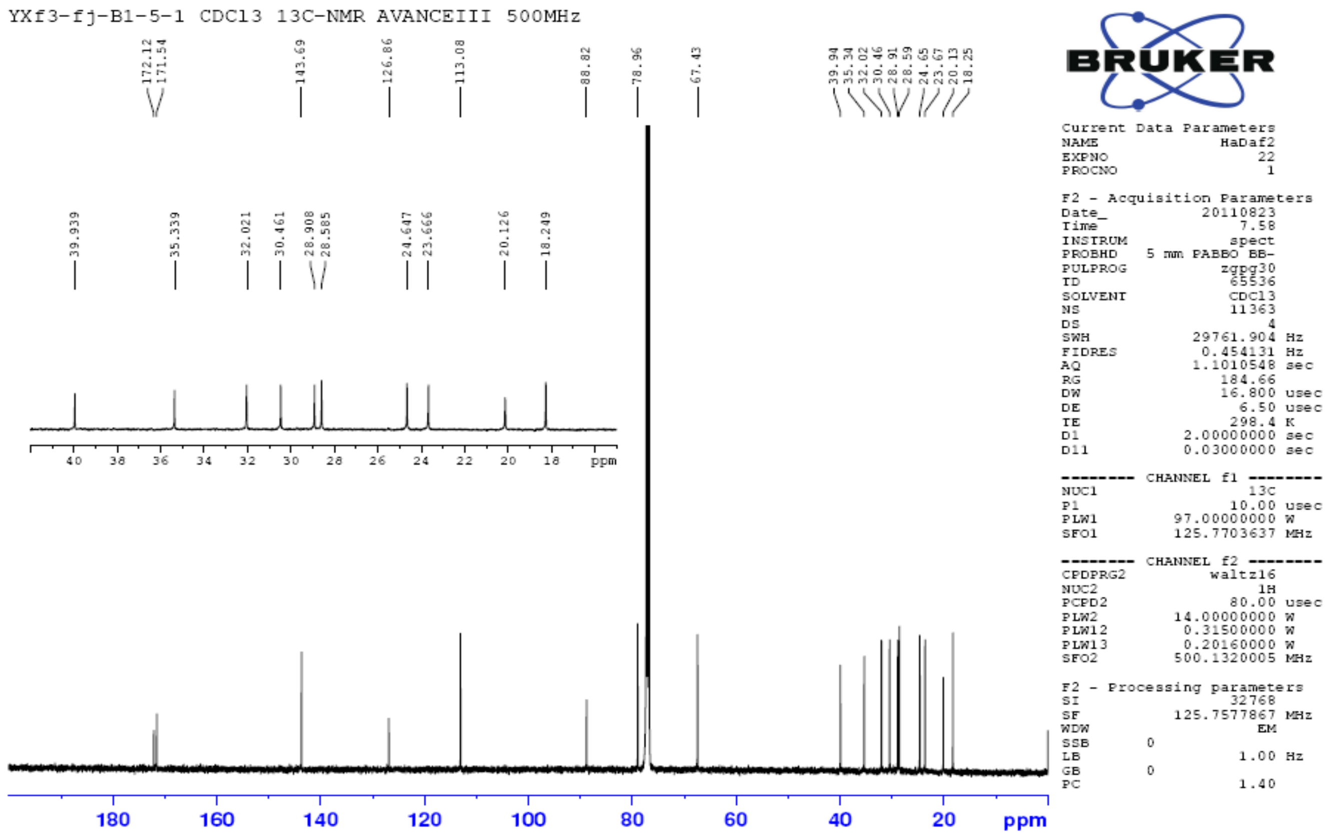

Figure S11. HMQC spectrum of compound **1** in $CDCl_3$

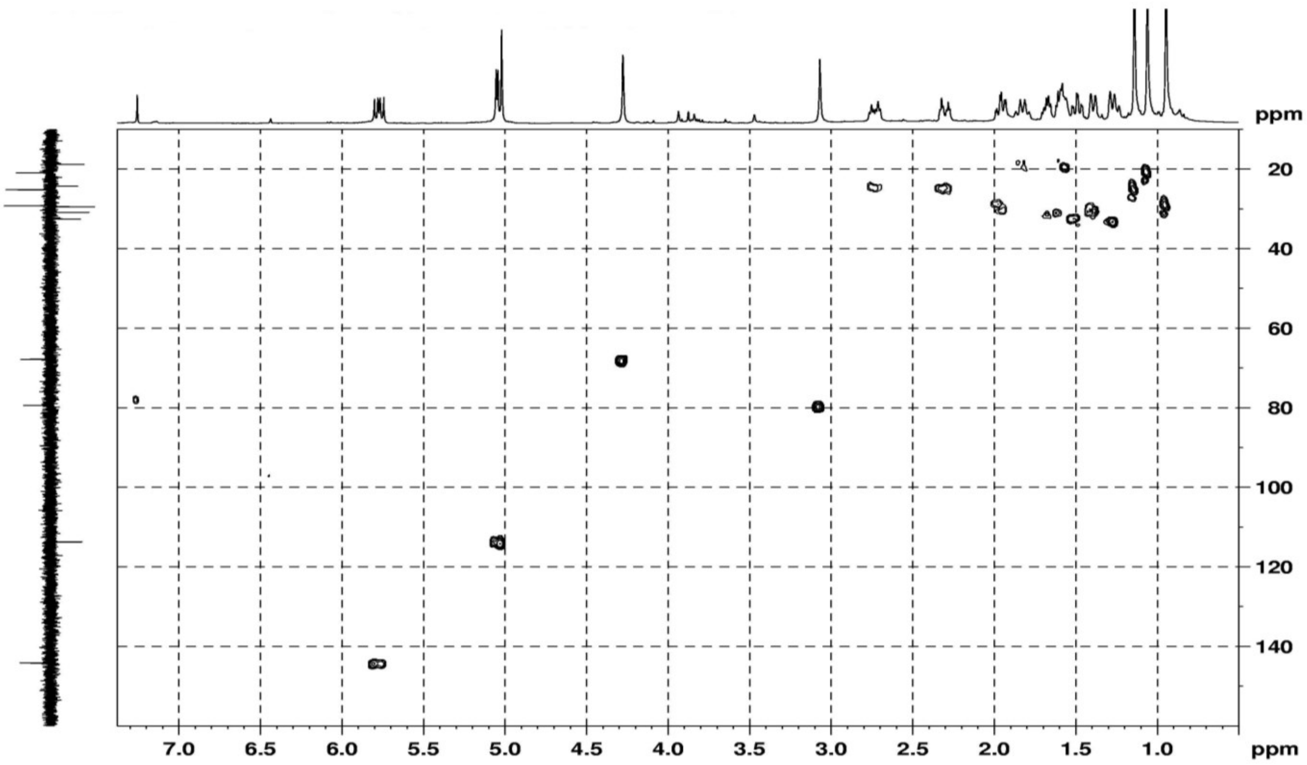

Figure S12. ^1H-^1H COSY spectrum of compound **1** in $CDCl_3$

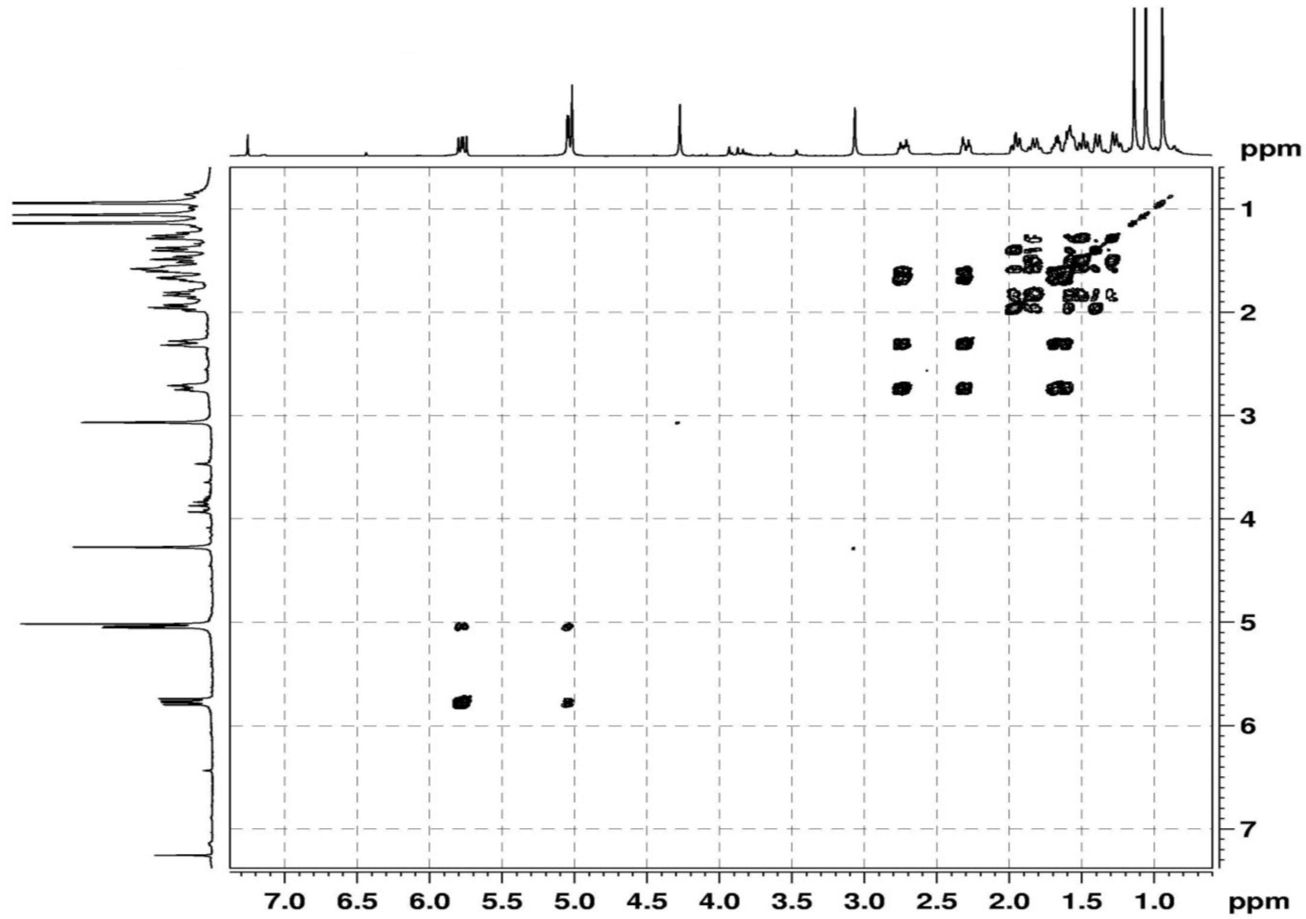

Figure S13. HMBC spectrum of compound **1** in $CDCl_3$

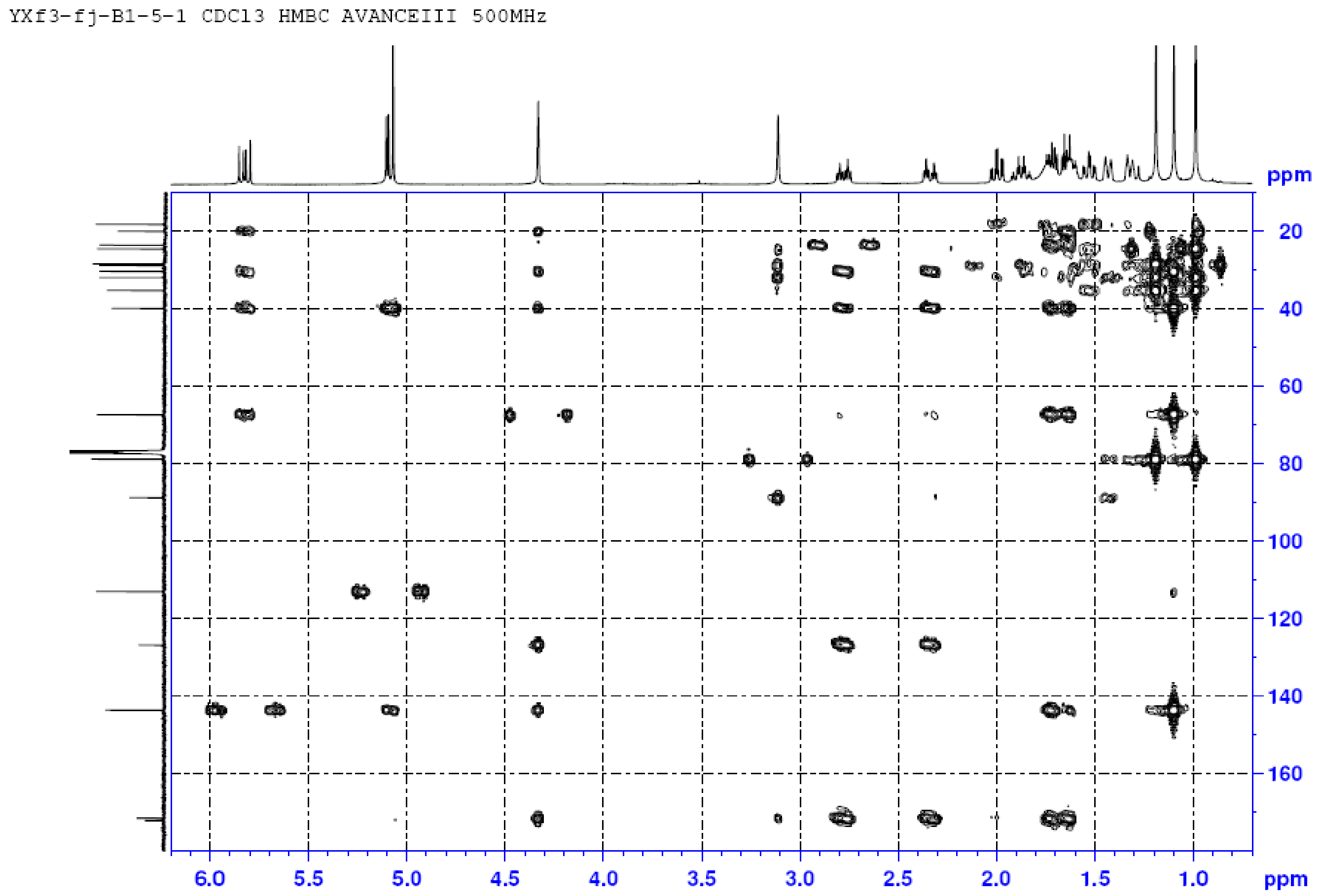

Figure S14. Enlarged HMBC spectrum (δ_C 177-167 ppm, δ_H 5.0-0.9 ppm) of compound **1** in $CDCl_3$

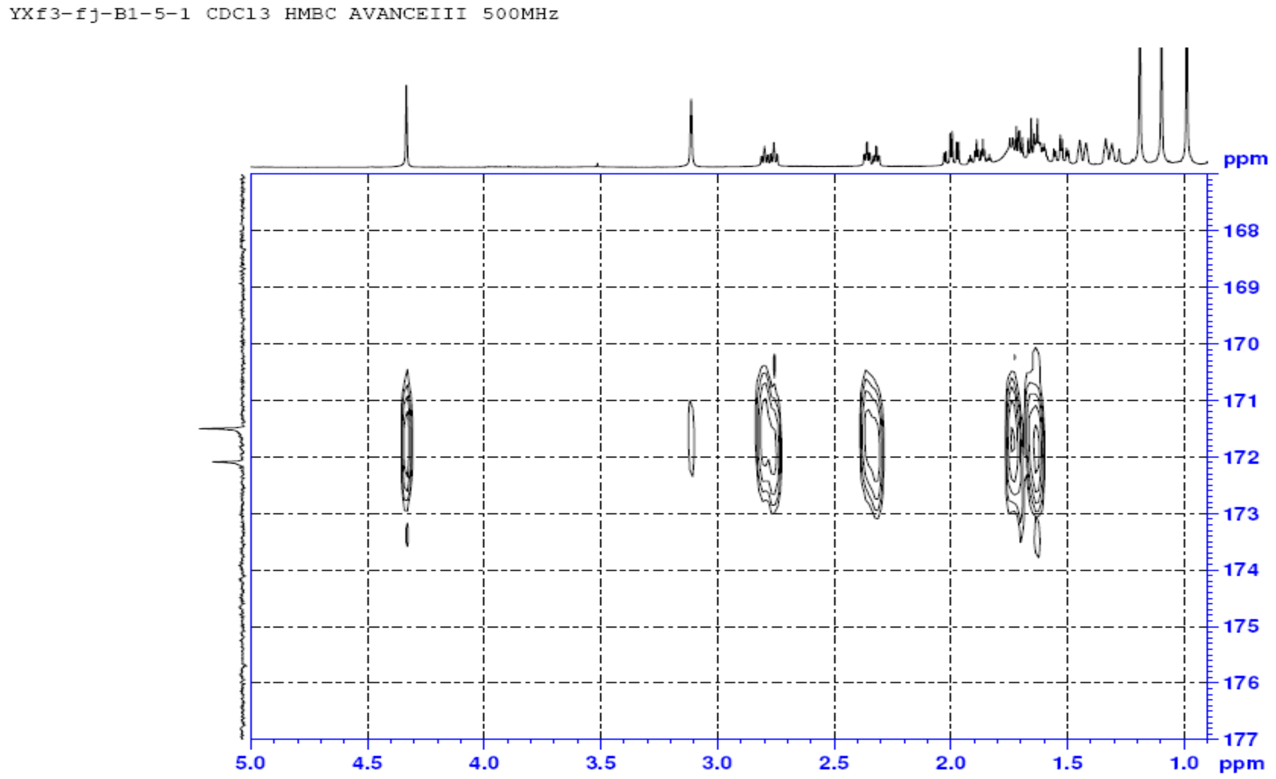

Figure S15. HRESI-MS spectrum of compound **1** in MeOH

Qualitative Analysis Report

**Data Filename**	YXF3-FJ-B1-5-1.d	**Sample Name**	YXF3-FJ-B1-5-1
**Sample Type**	Sample	**Position**	P1-A6
**Instrument Name**	Instrument 1	**User Name**	
**Acq Method**	TEMP2.m	**Acquired Time**	11/26/2010 3:40:55 PM
**IRM Calibration Status**	Some Ions Missed	**DA Method**	Default.m
**Comment**			

User Spectra

Spectrum Source	Fragmentor Voltage	Collision Energy	Ionization Mode
Peak (1) in "+ TIC Scan"	170	0	Esi

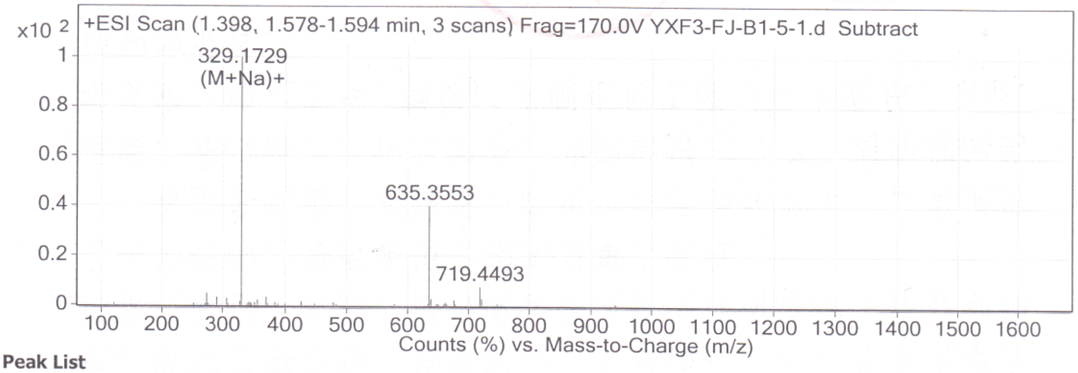

Peak List

m/z	z	Abund	Formula	Ion
329.1729	1	778087	C18 H26 Na O4	(M+Na)+
329.399		51889		
330.1758	1	137615	C18 H26 Na O4	(M+Na)+
635.3553	1	309541		
636.3587	1	109646		
719.4493		57309		

--- End Of Report ---

Calc. $(M+H)^+$ 307.1904

$(M+Na)^+$ 329.1723

Figure S16. ^{1}H NMR (500 MHz, acetone-d_6) of compound **6**

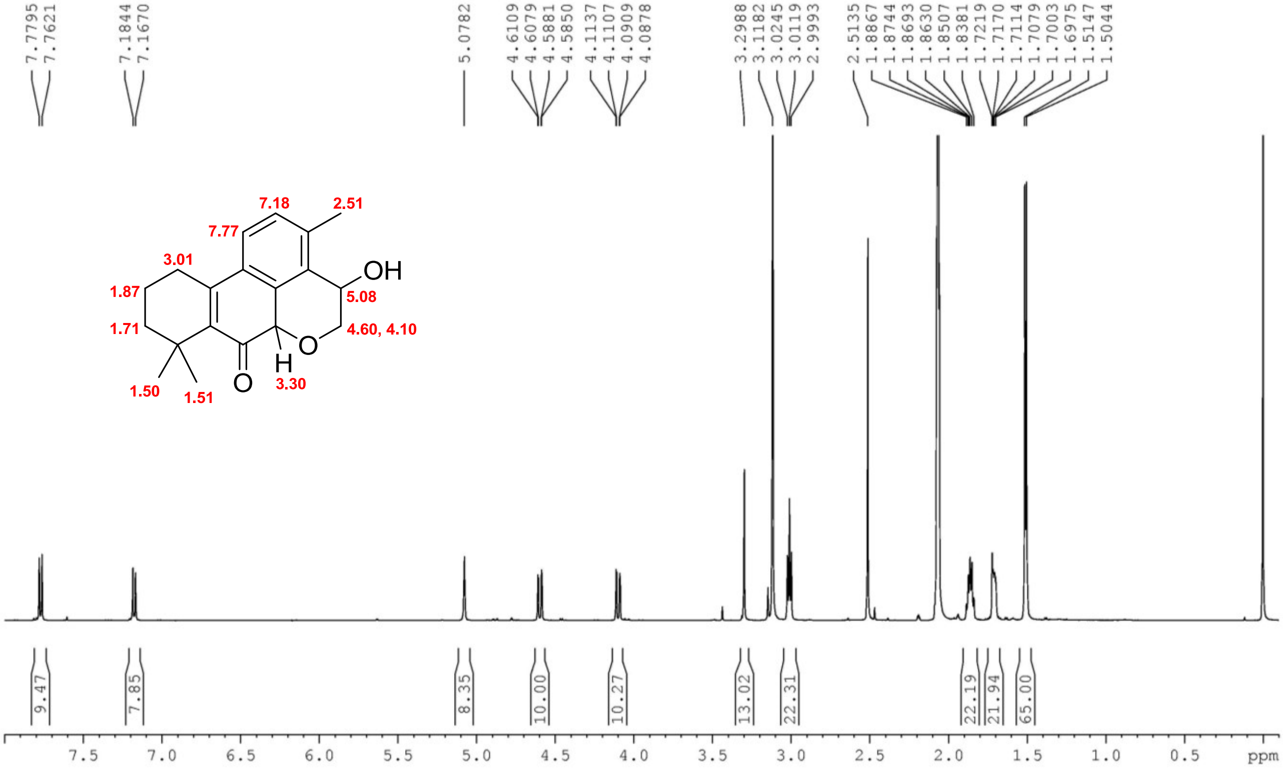

Figure S17. HRESIMS spectrum of compound **6**

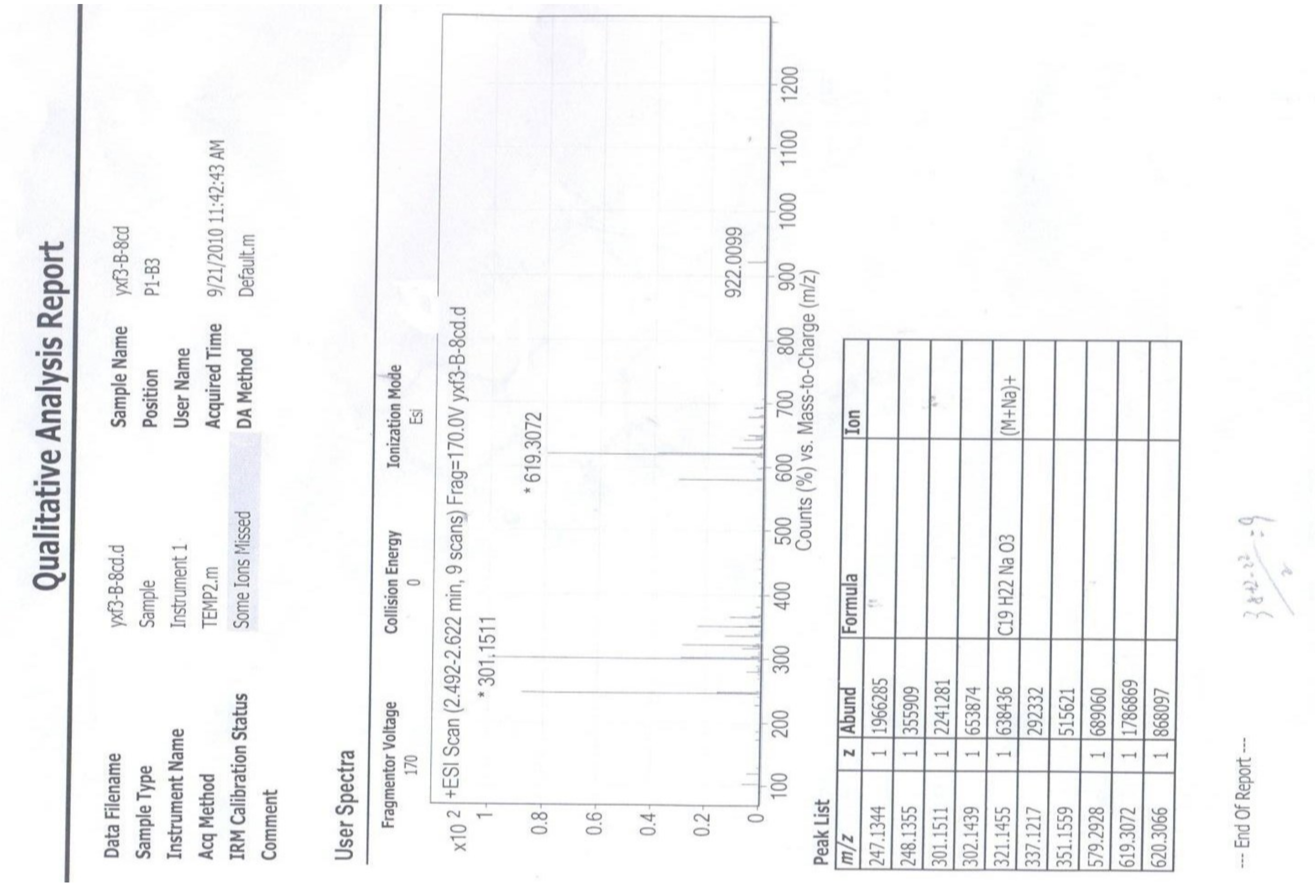

Figure S18. ^{1}H NMR (500 MHz, acetone-d_6) of compound **7**

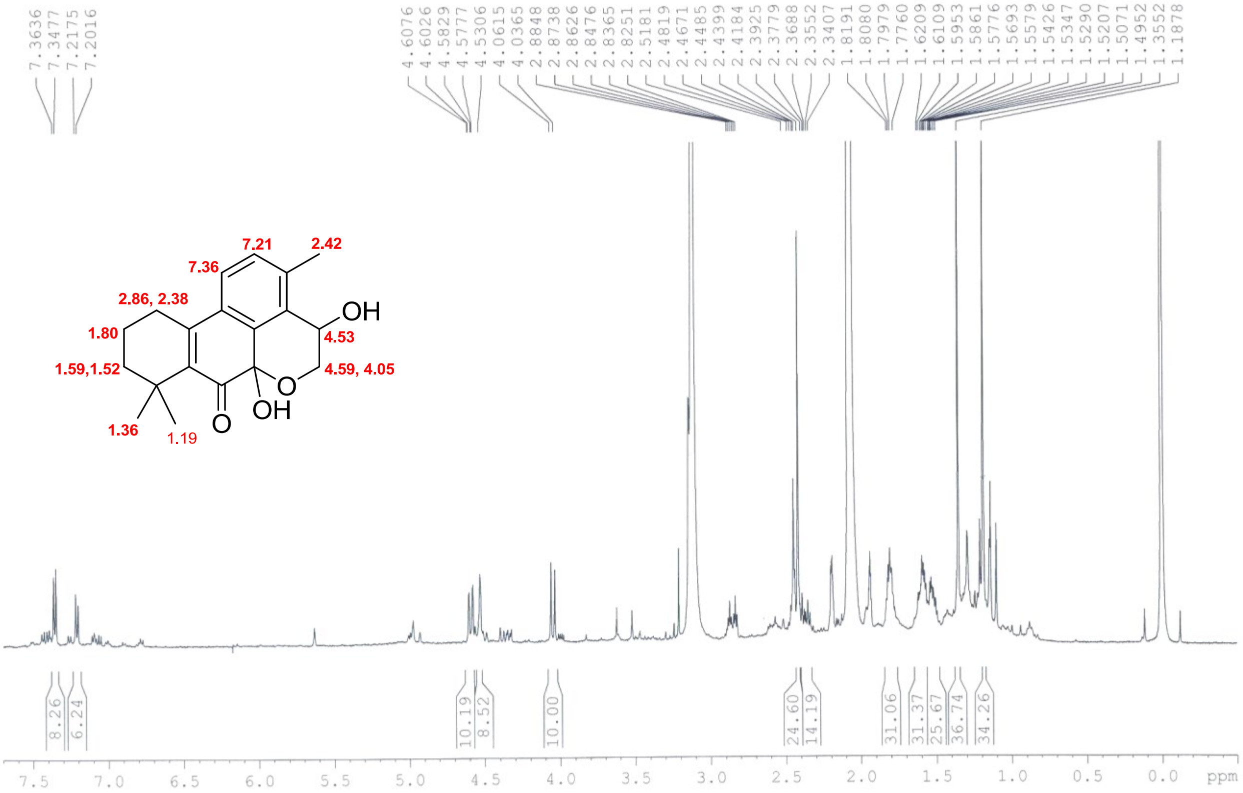

Figure S19. HRESIMS spectrum of compound **7**

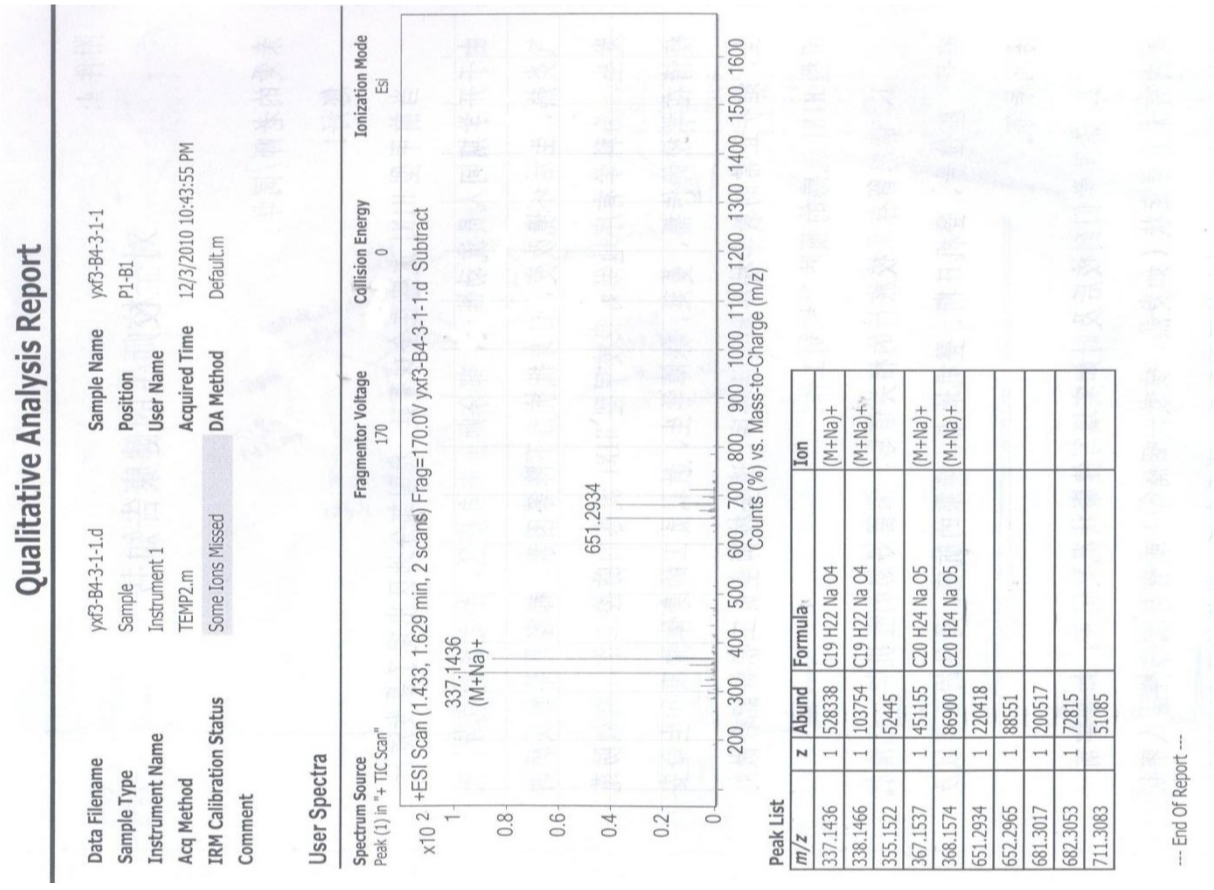

Qualitative Analysis Report

Data Filename	yxf3-B4-3-1-1.d	Sample Name	yxf3-B4-3-1-1
Sample Type	Sample	Position	P1-B1
Instrument Name	Instrument 1	User Name	
Acq Method	TEMP2.m	Acquired Time	12/3/2010 10:43:55 PM
IRM Calibration Status	Some Ions Missed	DA Method	Default.m
Comment			

User Spectra

Spectrum Source	Fragmentor Voltage	Collision Energy	Ionization Mode
Peak (1) in "+ TIC Scan"	170	0	Esi

Peak List

m/z	z	Abund	Formula	Ion
337.1436	1	528338	C19 H22 Na O4	(M+Na)+
338.1466	1	103754	C19 H22 Na O4	(M+Na)+
355.1522	1	52445		
367.1537	1	451155	C20 H24 Na O5	(M+Na)+
368.1574	1	86900	C20 H24 Na O5	(M+Na)+
651.2934	1	220418		
652.2965	1	88551		
681.3017	1	200517		
682.3053	1	72815		
711.3083		51085		

--- End Of Report ---
